# “That’s a child, it’s not a diagnosis.” What can paediatricians learn from medical humanities?: a mixed methods study

**DOI:** 10.15694/mep.2019.000130.1

**Published:** 2019-06-12

**Authors:** Elinor Thomason, Barbara A. Jack

**Affiliations:** 1 Alder Hey Children's Hospital; 2 Edge Hill University

**Keywords:** Medical humanities, paediatrics, communication, empathy, reflection, postgraduate teaching.

## Abstract

This article was migrated. The article was marked as recommended.

**Objective:**The aim of this study was to evaluate UK paediatric specialist trainees’ perceptions of a medical humanities teaching session on their communication and empathy skills.

**Methods:** A medical humanities session was incorporated into a teaching programme for 19 doctors in their first three years of paediatric training. Using set questions, participants discussed themes of communication, empathy, ethical issues and language. A qualitative methodology was adopted for the evaluation. All doctors who undertook the session were invited to join in a digitally recorded focus group and nine participated. Thematic analysis of the transcript was undertaken by two researchers to identify and code key themes. Six months post-course all participants were invited to complete an online survey looking at the longer-term impact of the session and five responded.

**Results:**Coding of the transcript identified two key themes that participants felt the session added to their usual teaching: i) communication and ii) reflection.

**Conclusion:**Literature-based teaching for junior paediatric doctors was well received and valued by participants and adds to standard teaching. It provides a platform for consideration of the parental perspective and communication (in particular the use of language) as well as providing structured time for reflection on clinical experiences.

## Introduction

The value of medical humanities teaching is well recognised and it has been incorporated into many undergraduate medical curricula in the United States of America (USA) and Durham University in the United Kingdom (UK) but there is very little evidence in the literature of its use in postgraduate medical training in the UK ( Macnaughton, 2000). Medicine itself is an art, not purely a science, and it is being increasingly recognised that many aspects of being a good clinician cannot be taught only from medical textbooks. Medical humanities teaching embraces the fact that it matters not only what a doctor knows or can do, but who they are as a person ( Macnaughton, 2000; Mill, 2006). It is suggested that incorporating humanities into medical education promotes reflective skills, improves communication skills and empathy, professionalism ( Wald *et al*., 2018; Mann, 2017) and has been shown to help make medical students more holistic doctors ( Macnaughton, 2000; Ousager and Johannessen, 2010). Medical humanities can enhance medical student education to ensure that they are not just ‘trained’, but also ‘educated’ and additionally can promote critical thinking, analytical and narrative skills ( Macnaughton, 2000; Rudolf and Storr, 2003).

Medical humanities has been shown to improve medical student skills in two key areas: communication and empathy ( Bleakley, 2015; Evans, 2003; Lancaster *et al*., 2002). Bleakley (2015) notes that medical students who did an undergraduate humanities degree in the USA performed the same as those who did undergraduate science degrees in all areas, with the exception of communication where they outperformed their peers ( Bleakley, 2015).

As a branch of medical humanities, literature has many benefits to offer doctors in their training. Literature opens up new worlds, cultures and perspectives to the reader and helps doctors step out of their own background and culture to understand those of their patients ( Macnaughton, 2000). Lancaster *et al*., (2002), using literature in medical education, found that students valued most the insights they gained into their patients’ experience of illness. Other authors agree and outline the main ways in which the study of literature in medicine is beneficial: improving communication skills, to stimulate and encourage an enduring sense of wonder at embodied human nature, to help doctors to understand the lives of sick people and the impact of what they, as doctors, do ( Evans, 2003; Lancaster *et al*., 2002).

However, there are many barriers to including medical humanities in medical training, not least the time and space it requires in the packed curriculum ( Horton, 2019). The study of literature is time consuming, in both the time taken to read resources and time to discuss what has been read. Some students do not enjoy reading for pleasure or study and may disengage. Some undergraduate courses have tried to overcome this by offering medical humanities or literature teaching as an optional special study module for which students self-select to utilise this teaching. At postgraduate level, with all the pressures of clinical work and revision for postgraduate exams (where the emphasis is on clinical and scientific aspects of medicine) there is little time available for this.

In postgraduate paediatric training in the UK there is very little, if any, medical humanities teaching. Rudolf and Storr (2003) describe a session they organised for senior paediatric trainees where they used literary sources as part of a Masters module at Leeds University. They note that use of the arts can widen the paediatrician’s perspective on the patient experience and our profession as a whole, in particular how we as doctors are portrayed. Those present found the session to be both enjoyable and effective. They raise an important question about the use of literature in postgraduate paediatric training: “If reading can increase our sensitivity to ourselves and the impact we have on patients, along with giving us a better understanding of illness and how it affects our young patients, might there be a value in formally incorporating reading into our professional development?” ( Rudolf and Storr, 2003, page 636).

## Methods

A teaching session using a short video clip of a parent discussing her son’s diagnosis of Down Syndrome from a BBC news interview and excerpts from literature (including blogs written by parents, non-fiction and fiction) was developed. These sources were read or viewed and the participants used set questions as a starting point for further discussion in small groups. The key points from these discussions were fed back to the whole group. The sources used and questions given were designed to encompass themes of communication, paediatric ethical issues, empathy and language as these have been previously reported to be areas where medical humanities teaching is most useful ( Evans, 2003; Lancaster *et al*., 2002). Part of this session was piloted in a departmental teaching session with a range of specialist nurses, paediatric and neonatal doctors of different grades. It was then amended based on feedback from this and delivered by the lead researcher to 19 ST1-3 paediatric trainees in a regional teaching session.

All session participants were invited to attend a digitally recorded focus group immediately following the session and nine of the participants agreed to contribute (response rate 47%). All who agreed to participate were given information about the focus group and the opportunity to ask any questions. Those who agreed to participate provided written consent. The focus group approach is widely used in health research and is appropriate as it allows for exploration of the participant’s opinions and experiences, encourages discussion and debate and allows for clarification of comments ( Kruger and Casey, 2014). The focus group was digitally audio recorded, moderated by the researcher who carried out the session and lasted for thirty minutes. A semi-structured interview schedule (see Appendix 1) guided the discussion using open ended questions and participants were also encouraged to provide more detail and give specific examples of clinical practice. A single focus group was used due to time constraints. The reporting of this study has adhered to COREQ guidelines ( Tong *et al*., 2007). Approval to undertake the study was granted by Health Education England Research Governance Committee.

A qualitative methodology was adopted for the evaluation and thematic analysis of the transcript of this focus group was undertaken by two researchers to identify and code key themes. We attempted to achieve rigor by using multiple coders and keeping detailed records of data analysis activities, ensuring auditability. Two researchers analysed the work separately, reducing the potential for bias in interpretation. They subsequently deliberated and agreed on the themes and coding definitions with no disagreements and together checked the consistency and accuracy of the interpretations that had been made.

Six months after the session all participants were contacted again and asked to complete an anonymous online survey looking at the longer-term impact of the session on their clinical practice. Five of the trainees completed this survey. The questions asked on this survey are listed in Appendix 2.

The learning objectives for the session were drawn from the Royal College of Paediatrics and Child Health (RCPCH) level 1 curriculum and focused on curriculum items that were difficult to evidence from clinical practice or standard teaching, for example ‘begin to develop strategies to communicate sympathetically with parents’ (RCPCH, 2018). All 5 trainees who completed the survey agreed that the session had met their curriculum requirements. However, following this, the RCPCH has brought in a new ‘RCPCH Progress’ curriculum, but this literature-based teaching session would still cover multiple domains of the new curriculum.

## Results/Analysis

The session was well-received by participants and most asked to have similar sessions incorporated into their future training and gave suggestions for topics they wanted to see included in these. There was a perceived absence of any participants having ever had any medical humanities resources or teaching in previous undergraduate and postgraduate medical training. The thematic analysis generated a series of codes which were grouped into two thematic categories [ Table 1]; 1) reflection on learning included sub-themes of considering the parental viewpoint, reflective practice and becoming a holistic doctor and 2) communication, including sub-themes of language and the impact of the session on future practice. Examples of quotations are included to illustrate the key points, with each being identified by the participant number and gender of the focus group participants.

**Table 1.** Thematic categories and constituent codes of trainee perceptions of literature based medical humanities post-graduate paediatric teaching

Reflection on learning	Communication
Considering parental perspective	Language
What parents remember	
Becoming holistic doctors	
Reflective practice	Impact on future clinical practice

### Reflection on learning

Many participants reflected on the fact that they had never previously considered the parental perspective as they did in this session and parents are often spoken about in professional conversations in a negative way:

*“I feel like a lot of the time when you’re talking between doctors about parents, it’s pretty negative actually and we usually dismiss, ‘oh they’re being really annoying’..whereas, in this talk, we’ve all been very much like thinking about it from the parents’ point of view, so it’s been useful. But I also think we definitely don’t do that enough.”* (Female participant 2).

*“Going through these in the way we did is the most I’ve ever thought about how it must be from the parents’ side, so even though we all think that’s our strength.”* (Female participant 1).

Participants highlighted, with examples from clinical practice and the excerpts used in the session, that the parents’ agenda is often different from the healthcare professional’s perception of what the parents want to get out of the encounter. A few participants commented that the way some parents would prefer to have information given (particularly in relation to breaking bad news) differs from the set way that they have been taught to do this. The session caused many participants to reflect on the different perspectives that the doctor and the parent are coming from, with the doctor often focusing on the biological or diagnostic considerations of the disease but the parent’s main focus being on how this affects their child and their family:

*“They’re not interested in the ins and outs of what a syndrome is, what it means, necessarily, they are more looking for support, which you can forget about sometimes.”* (Female participant 3).

Many participants commented that some of the things that parents take away from consultations, in particular use of language, are not something they’d previously considered. They discussed the fact that the doctor feels different emotions in these situations, for example breaking bad news, to the parents but the way that the doctor behaves in response to that can influence the parental response to what is said to them. Multiple participants commented that the consultation ends for the doctor but for the parents they then have to move on with their lives with the information they’ve been given:

*“It’s not necessarily what you told them; it’s how you did it and how it made them feel at the time and therefore how they sort of carried on with their lives from that really.”* (Male participant 1).

A recurring theme that participants brought up was how this session helped them to become more rounded or holistic doctors. One participant linked this with becoming more empathetic and many commented that this was missing from usual teaching programmes. The participants felt that this medical humanities teaching session was valuable as it covered different skills than they were usually taught on and these, in particular considering empathy towards parents (and patients), made them more well-rounded doctors. One felt that this translated into better patient care:

*“If you can’t communicate that back to families and parents or colleagues, then you’re not really fulfilling your role as a holistic doctor and that’s probably the most important side and that’s also the thing that families and parents and patients will actually remember about you.”* (Male participant 1).

While the session was designed as a teaching session, the trainees felt that it actually provided invaluable opportunities for group reflective practice that they didn’t usually have opportunity for. Participants commented that having the opportunity to discuss difficult situations and learn from others allowed them to develop and explore their humanity as doctors, rather than being merely practitioners. A few participants discussed how some of the topics covered were difficult and emotional but being able to openly discuss them with other trainees provided comfort and support:

*“We’re all a bit, sort of, damaged because we just see so much sad, horrible stuff.”* (Female participant 2).

*“Also, to note that other people find things difficult.. lots of people haven’t been in that position, so it’s a little bit comforting.”* (Female participant 3).

Many participants reflected on how they were unaware of the impact of their words or their actions on the families of the patients they treat:

*”It’s not often you think about just the impact of what you’re doing, you know, thinking about the other side of the fence as it were.”* (Male participant 1).

Some trainees commented that to take their learning from this session further in the future, they planned to use parent-written blogs and other medical humanities resources to enable them to improve their communication and empathy skills and develop as more well-rounded and holistic doctors.

### Communication

The single most common theme identified from the discussion was the use of language in patient and parent encounters. Trainees discussed how they had previously been unaware of the significance of some of the language they used in consultations and the enduring impact this can have on families. There were many references to a video clip that was discussed, where a mother highlighted the impact that the doctor’s use of the word ‘sorry’ had on her experience of being told her son has Down Syndrome. Many participants commented that they had not realised how the use of certain words or phrases (that they may not consciously be aware of using) can impact parents and children for a long time after the consultation:

“ *A lot of parents pick up on very small use of language.. it’s sometimes just one phrase that you can use that can just slightly stick in a person’s mind and they will then remember that ongoing, so you have to be very careful with the words that you choose to use.”* (Male participant 1).

Some trainees reflected on the use of language between professionals, as well as in parent consultations, and how this can influence how we view the patients. One participant pointed out that our language as professionals can reduce children to diagnoses rather than viewing them as individuals:

*“I don’t know about you but quite often on ward round, we’ll say, ‘oh that’s the girl with CF’ or, ‘that’s the girl with Down’s Syndrome’, whereas it isn’t. That’s a child, it’s not a diagnosis, which I think is important.”* (Female participant 4).

One participant referred to a recent high-profile media case at the hospital and discussed how failure in communication had played a part in the legal and media issues that ensued. Other participants picked up on this and discussed how as paediatricians we think that we are good at communicating but this case, and the themes brought out from the teaching session, highlight that this may not in fact be the case, but is a very important area for paediatricians to improve on:

*“I think communication seems to be a lot more in the media than it used to be.. where there’s obvious communication breakdown between the doctors and the parents so it’s an area we do need to sort of improve in as a speciality.”* (Female participant 4).

Through the focus group, many trainees reflected on their learning from the session and spontaneously commented on how this would impact their future clinical practice, mainly relating this to communication. Some participants also commented that the pressures of clinical work impacted on their ability to communicate as effectively as they would like to. One participant considered that she had previously often considered what she would say in a consultation prior to going in, but hadn’t thought about what she would not say, which she now felt was important in light of some of the parental accounts that had been considered. Another participant commented that improving communication skills is an ongoing process which is never complete:

*“There’s a way as well where you won’t always have the same situation every time and you might have to adapt what you say to different parents in different contexts and it’s something you will never be able to tick off and say, ‘I’ve learnt this.’ It’s something that you’ll do until the day you retire.”* (Male participant 3).

### Survey responses

6 months after the session took place, an electronic online survey was sent out to all who attended the session and this was completed anonymously. All five trainees who completed the survey said that they had changed their clinical practice following the session, but most were unsure if they had changed their use of language as a result of the session, despite this being the most-discussed area of change within the focus group. All five survey participants, however, did say that they have changed how they break bad news and feel they have more empathy towards children with disabilities and their families. The follow up survey responses indicated that all five trainees who completed it had gone on to look at medical humanities resources following the session and two had subsequently incorporated them into their own teaching for junior doctors and medical students. Consideration of the patient and parental perspective was the main theme of the session commented on by participants in the survey, suggesting that this was the key message they took home and continued to reflect on following the session:

*“It was good to remember that the person on the other side of the interaction comes from a different background to you, and that the interaction usually matters a lot more to them.”* (Survey participant 1).

*“Great awareness raising activity, helped me appreciate the patient’s perspective more than previously.”* (Survey participant 2).

## Discussion

The implementation of this literature-based medical humanities in postgraduate paediatric teaching was both well received and valued by those who attended the session. The two main themes that participants discussed in the focus group were that the session had improved their communication skills and, in their reflection on the learning, they considered the parental perspective which demonstrated empathy. Both communication and empathy have been found in previous work to be areas where medical humanities has its greatest benefit ( Evans, 2003; Lancaster *et al*., 2002). The participants themselves noted the link between what they had been learning and discussing and how it made them more rounded and holistic as doctors, rather than purely scientists, which is in line with previous research on medical humanities teaching ( Ousager and Johannessen, 2010).

One unexpected finding from the focus group was the opportunity for participants to reflect on their own clinical experiences and discuss how they will respond to situations in the future (i.e. how they would use the learning from this session in clinical practice going forwards). This constitutes deep learning and the trainee’s ability to translate this knowledge to real world (past and future) situations makes it more likely to impact on their future practice ( Gordon and Evans, 2010). This was evidenced in the follow up survey when all five students agreed that this session had changed their clinical practice in some areas. As very few trainees responded on the survey to the question about whether it had changed their communication skills in clinical practice, is not clear whether the effect on communication skills persisted long-term, however, trainees did comment that it had changed the way they break bad news (which is a communication skill). Perhaps narrowing the definition of communication skills in the survey or asking trainers if they had noticed perceptible changes in the way trainees communicate may have provided more information on this.

The benefit of reflective practice is well recognised and is a General Medical Council requirement for doctors (AOMRC, 2018). Bleakley (2015) has highlighted the need for doctors to be able to tell their own stories (narrative) to other doctors and the benefit this has on them which is particularly relevant in a profession with increased rates of suicide and depression compared to the general population (Centre *et al*., 2003). Participants in this session perceived it to be a safe place to discuss and reflect on their personal experiences of clinical encounters. In order to continue to benefit from the group reflection aspect of the session, future sessions would need to be designed taking into consideration the group composition and dynamics in advance.

There are a number of limitations of this study. As with much qualitative research, the sample size was small and as the teaching session was only implemented in a single regional teaching day the results are only applicable to this setting and may not be generalizable across all paediatric trainees. Incorporating the session into a mandatory teaching day ensured that the session was not only attended by those interested in medical humanities teaching, but all trainees were expected to attend. There is always the possibility of researcher bias in qualitative research and this is a possibility in this study as the lead researcher is a paediatric trainee who had past working relationships with some trainees and will have future working relationships with them. This may have led to participants reporting what they felt the researcher wanted to hear, although they were specifically asked not to do this. Incorporating the anonymous follow-up survey was designed to allow the opportunity to provide additional feedback. The comments given here were similar to those in the focus group, suggesting that the participants were reporting their actual views. Some of the topics covered were emotionally difficult and participants may have not wanted to ‘lose face’ among other trainees and therefore may have self-censored some of their views. A strength of the study was the longitudinal aspect through use of the follow up survey to review the longer-term impact of the session on the participant’s communication and empathy skills. A second focus group would have been preferable to explore this, but this was impractical to arrange as not all the same trainees attend each regional teaching session.

## Conclusion

This study has demonstrated that it is possible to incorporate literature-based teaching into postgraduate paediatric teaching in a manner that does not take up a lot of time both within the session itself or a requirement for a lot of pre-reading. Participants reported that the session was valuable and enjoyable and enabled them to meet their RCPCH curriculum requirements. It adds to standard approach to teaching postgraduate medical staff which normally includes formal lectures on clinical conditions and their management. The session met its objectives of improving participants’ empathy and communication skills. The session provided structured time for reflection on clinical experiences, such as cases with complex ethical considerations and families with whom the communication had been challenging, which was highly valued by participants. This was a limited study but the longitudinal aspect highlights that this has impacted on the participant’s future clinical practice. Within medical education there should be a move away from focusing only on clinical knowledge and training (not just education) should promote the development of holistic doctors and medical humanities teaching can be used to achieve this.

## Take Home Messages

• Literature based medical humanities teaching was well-received and valued by paediatric trainees and added to standard teaching programmes.
• It was particularly useful for discussing communication and consideration of the parental perspective.
• It provides an opportunity for reflection on clinical cases seen and how to improve future practice.
• The longitudinal element suggests this approach is valuable for improving patient care and developing well-rounded or more ‘humane’, holistic doctors.

## Notes On Contributors

Dr Elinor Thomason, MB ChB, MRCPCH, PGCert is an ST7 paediatric trainee in the North West (Mersey) deanery who completed this research as part of a project for a Medical Education Fellowship with Health Education England (North West). ORCID ID https://orcid.org/0000-0003-3039-279X

Barbara Jack PhD, MSc, BSc(econ), RN, RNT, PGD is a Professor of Nursing at Edge Hill University.

## Appendices

### Appendix 1: Semi-structured questions for focus group

1. Have you ever had medical humanities teaching or used any medical humanities resources previously?
2. What did you think of the session?
3. What do you think about the structure and facilitation of the session?
4. Did it meet your learning needs and learning outcomes?
5. Did you learn anything you didn’t expect to?
6. Did you not learn something that you expected to learn?
7. How will having done this session change your behaviour and practice?
8. What did you think of the use of different kinds of media and resources?
9. Would you want to do a similar session again in the future?
10. Do you think medical humanities offers something you don’t usually get in teaching?
11. Do you have any other comments?

### Appendix 2: Survey questions

1. Have you ever had any medical humanities teaching (besides the STEP 1 teaching session)?

2. Have you looked at any medical humanities resources since the session?

3. Have you used any medical humanities resources in your own teaching since this session?

4. Have you changed or altered the language you use when talking to patients or parents since attending this session?

5. Did attending this session improve your empathy towards children with disability and their families?

6. Please rate how much you agree/disagree with the following statements [options given: Strongly agree, agree, neither agree nor disagree, disagree, strongly disagree]:

A) The teaching session gave me a better understanding of some of the challenges facing families of children with disabilities.

B) This teaching made me change the way I break bad news to children and families.

C) I learnt something that I was not expecting to learn in the session.

D) I feel that I did not benefit from attending the session.

E) The session helped me to achieve some of my MRCPCH curriculum requirements.

F) My clinical practice did not change in any way after attending this session.

G) I would be keen to attend further medical humanities sessions in the future.

H) I have changed the way I communicate with children with disabilities and their families following this session.

I) The session has made me consider difficult ethical scenarios from the parents’ perspective in a way I hadn’t before.

7. Is there any other impact that this session has had on your clinical practice or attitudes besides those already mentioned? Please elaborate.

8. Do you have any further comments?
