# Supplementary material for: “That’s a child, it’s not a diagnosis.” What can paediatricians learn from medical humanities?: a mixed methods study
Source: MedEdPublish (2016). 2019 Jun 12;8:130. [Version 1] doi: 10.15694/mep.2019.000130.1 (PMC10712540; doi:10.15694/mep.2019.000130.1)
Supplement: Peer review history

# Reviewer report: Reviewer response for version 1

**DOI:** 10.21956/mep.19866.r30017

**Published:** 2020-05-02

**Authors:** Subha Ramani

**Affiliations:** 1 Harvard Medical School, Brigham and Women's Hospital

This review has been migrated. The reviewer awarded 4 stars out of 5 I enjoyed reading this paper and messages highlighted by the authors resonated with me as a clinician and educator. This is an important area for research, more institutions are including humanities teaching in their medical education. with burgeoning technology and decreased in-person time spent by trainees with patients/families, patient care is frequently deconstructed into a list of tasks for the day. I commend the authors for including this curriculum and building in time for reflection.The paper would add value to all educators leading and teaching communication skills, humanism in medicine. It is well known that communication breakdown issues are more frequently the cause of patient complaints, dissatisfaction and non-adherence.The title reflects the rest of the paper.Abstract is reasonable but could give a few more details especially under results.Introduction- gives a good overview of humanities in medical education. I would suggest a more explicit statement of the problem and the gap in literature. As it stands, I cannot grasp whether this study aims to provide results which are different from other investigators. I also do not see explicit study questions or objectives.Methods- the study methodology is appropriate for the aims, though I mentioned before that clear study questions are needed.Study setting and who the participants are can be described in more detail for readers to understand the context.Recruitment and sampling are describedMore details on data analysis (coding and theme abstraction) would be useful. I am also not seeing any mention of reflexivity. Having said all that, one focus group may be inadequate and study sample is small- even for a qualitative study. We cannot be assured that we have data saturation.Results- very well written. The themes make intuitive sense and the quotes appear to lend meaning to the investigator interpretation and reporting.The themes themselves are well described.The post-survey does not add much value as it stands.Discussion- summary of findings, the unexpected focus on reflective practice, limitations are all well written and appropriate.The only challenge I see is whether these results are broadly applicable based on the participant number.Of the 9, we cannot be sure whether all expressed their opinions or it was a talkative few that drove the discussion.While I enjoyed the writing, the topic and the results, I see this as a pilot study with less rigor than needed.These results can certainly launch a rich and larger study. when I say larger, I mean depth rather than numbers. But we do need more focus groups.I hope the authors will view these comments as a motivator to further research as this topic is very important.

Reviewer Expertise:

NA

No decision status is available

# Reviewer report: Reviewer response for version 1

**DOI:** 10.21956/mep.19866.r30015

**Published:** 2019-10-04

**Authors:** Ken Masters

**Affiliations:** 1 Sultan Qaboos University

This review has been migrated. The reviewer awarded 3 stars out of 5 An interesting paper on a small mixed methods study on what paediatricians can learn from medical humanities. The Introduction gives a good background on the issue of the value of humanities in the medical curriculum, and the lack of research on its value in postgraduate medical education. The results are also useful, both to the institution and on a more general scale.There are, however, several issues in the writing of the paper that need to be addressed. I would really like to see a Version 2 of this paper that addresses these concerns.• No information about the setting/location of the study has been given. This, in addition to the educational context at the institution, is crucial for the paper.• Similarly, there is no information about the participants, apart from a rather short description “19 ST1-3 paediatric trainees in a regional teaching session.” • The opening paragraph is also confusing as it begins by speaking of participants in small groups, and then a pilot, and then the training. Are the 19 trainees the same participants mentioned in the earlier part of the paragraph, or was that a pre-pilot session? If different, who were those people? If the same, then it would be useful if the description of the process could be given in chronological order, and some information about the numbers and sizes of the small groups.• It would be useful if the sources used in the training session could be identified.• The authors refer several times to the study as “literature-based” but this does ignore the BBC video, which, from the results, appears to have had a strong impact. It appears that the study involved mixed media.• Although it is too late to make changes to the focus group questions now, I would strongly recommend that future focus group research questions are not so pointed and direct. (Some of them or even Yes/No questions). They really do need to be much more opened-ended, even in a semi-structured process.• Was software used in the coding? If so, it should be identified. If not, it would be best to say so specifically, especially as most qualitative coding today utilises some form of software.• Some demographic data of the focus group participants are required, particularly because the gender of the respondents is reported in the qualitative results.• At the end of the Methods, there is a mention of the LOs for the session. Is this the initial training session? If so, then it would better to place this at the beginning where the training session is described. (It can be referred to again here, but should be mentioned upfront).• From the opening sentence of the Results/Analysis, one might infer that there was some form of course/session evaluation performed. If so, some more detail of this evaluation (and numbers, not “most”) should be given. If not, then should be stated specifically to avoid confusion.• “There was a perceived absence of any participants having ever had any medical humanities resources” Was does “perceived” in this sense mean? Had they or had they not received medical humanities or pure humanities training prior to the session?• I realise that the low response rate to the survey means that meaningful statistical tests cannot be performed, but it would be useful to see the response numbers.• “One unexpected finding from the focus group was the opportunity for participants to reflect on their own clinical…” Why would this be unexpected? I would have thought that the main point of the session was exactly for them to reflect on and alter their approaches.So, a useful study, and the results point to further paths to be followed, but the writing of the paper needs to be addressed.

Reviewer Expertise:

NA

No decision status is available

# Reviewer report: Reviewer response for version 1

**DOI:** 10.21956/mep.19866.r30016

**Published:** 2019-06-13

**Authors:** Alice Fornari

**Affiliations:** 1 Donald and Barbara Zucker School of Medicine at Hofstra/Northwell

This review has been migrated. The reviewer awarded 4 stars out of 5 Thank you for contributing to the field of health humanities by including narrative and story telling a pediatric curriculum. Appreciate the appendices included to allow duplication of this effort at one's own institution with any group of clinical learners. Very useful to promote qualitative research core to this type of education. Your conclusions are very on target. The necessary simplicity of this curriculum to be realistic is valuable to medical educators who might experience barriers. Your reference list is useful too.

Reviewer Expertise:

NA

No decision status is available
